# Differential activation mechanisms of two isoforms of Gcr1 transcription factor generated from spliced and un-spliced transcripts in *Saccharomyces cerevisiae*

**DOI:** 10.1093/nar/gkaa1221

**Published:** 2020-12-24

**Authors:** Seungwoo Cha, Chang Pyo Hong, Hyun Ah Kang, Ji-Sook Hahn

**Affiliations:** School of Chemical and Biological Engineering, Institute of Chemical Processes, Seoul National University, 1 Gwanak-ro, Gwanak-gu, Seoul 08826, Republic of Korea; Theragen Bio Co., Ltd, 145 Gwanggyo-ro, Yeongtong-gu, Suwon-si, Gyeonggi-do 16229, Republic of Korea; Department of Life Science, College of Natural Science, Chung-Ang University, 84 Heukseok-ro, Dongjak-gu, Seoul 06974, Republic of Korea; School of Chemical and Biological Engineering, Institute of Chemical Processes, Seoul National University, 1 Gwanak-ro, Gwanak-gu, Seoul 08826, Republic of Korea

## Abstract

Gcr1, an important transcription factor for glycolytic genes in *Saccharomyces cerevisiae*, was recently revealed to have two isoforms, Gcr1^U^ and Gcr1^S^, produced from un-spliced and spliced transcripts, respectively. In this study, by generating strains expressing only Gcr1^U^ or Gcr1^S^ using the CRISPR/Cas9 system, we elucidate differential activation mechanisms of these two isoforms. The Gcr1^U^ monomer forms an active complex with its coactivator Gcr2 homodimer, whereas Gcr1^S^ acts as a homodimer without Gcr2. The USS domain, 55 residues at the N-terminus existing only in Gcr1^U^, inhibits dimerization of Gcr1^U^ and even acts *in trans* to inhibit Gcr1^S^ dimerization. The Gcr1^S^ monomer inhibits the metabolic switch from fermentation to respiration by directly binding to the *ALD4* promoter, which can be restored by overexpression of the *ALD4* gene, encoding a mitochondrial aldehyde dehydrogenase required for ethanol utilization. Gcr1^U^ and Gcr1^S^ regulate almost the same target genes, but show unique activities depending on growth phase, suggesting that these isoforms play differential roles through separate activation mechanisms depending on environmental conditions.

## Graphical Abstract

**Graphical Abstract:**
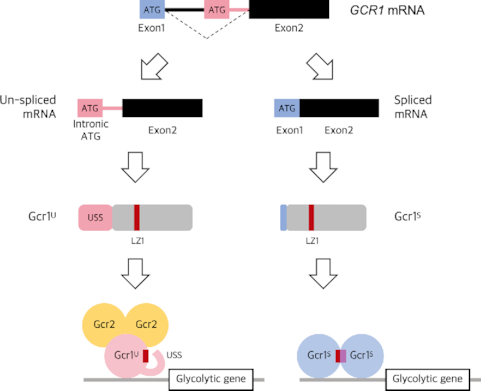
Gcr1^U^ and Gcr1^S^, generated from un-spliced and spliced forms of mRNA, respectively, act as different complexes to activate expression of glycolytic genes.

## INTRODUCTION

*Saccharomyces cerevisiae* is a Crabtree-positive microorganism that has a strong tendency to metabolize fermentable carbon sources such as glucose into ethanol via glycolysis and fermentation even under aerobic conditions (1). After consumption of fermentable carbon sources, carbon metabolism is switched to respiration to utilize ethanol, known as a diauxic shift (2). Therefore, regulation of glycolysis plays a central role in the carbon metabolism of *S. cerevisiae*. Expression of glycolytic genes is regulated by several transcription factors, among which Gcr1 is an important one (3–6). Gcr1 activates the transcription of many glycolytic genes by binding to CT boxes (CWTCC) in the promoter regions (7,8). In addition, Gcr1 lacking the C-terminal DNA binding domain can also activate transcription through interaction with the Rap1 transcription factor bound to promoters of glycolytic genes (9). Gcr2 works as a coactivator of Gcr1 without direct DNA binding (10) and both Gcr1 and Gcr2 can form homodimers through their leucine zipper (LZ) domains (11,12). It has been suggested that a heterocomplex consisting of a Gcr1 (monomer or dimer) and a Gcr2 dimer is involved in transcriptional activation of glycolytic genes, whereas the Gcr1 dimer is essential for the transcription of ribosomal protein (RP) genes (12,13). However, the role of Gcr1 in RP gene expression is controversial due to a growth defect accompanied by Gcr1 inactivation, which can indirectly affect RP gene expression (13). In fact, recent genome-wide ChIP-exo analyses showed that RP genes are not direct targets of Gcr1 (14).

The *GCR1* gene has an unusual feature of having a long intron, generating at least seven different spliced isoforms of mRNA by alternative splicing (15,16). Among the isoforms, only two were identified to produce in-frame proteins (16). The major spliced form is created by splicing out a 739-nt intron using the 5′ splice site (SS) GUAUGG and 3′ SS CAG, producing a Gcr1 protein of 789 amino acids, which we named Gcr1^S^. The other form exists in much lower abundance and uses 5′ SS GUAUGA and 3′ SS UAG located at 5-nt downstream and 17-nt downstream from the major 5′ SS and 3′ SS, respectively, to generate a Gcr1 protein of 785 amino acids, which we named Gcr1^A^. Accumulation of the spliced mRNAs increased in the later phase of cell growth around the diauxic shift, whereas un-spliced mRNA was observed as a major product during the early growth phase (16). A start codon located in the middle of the intron of the un-spliced mRNA is used as a translation start site, generating a Gcr1 protein of 844 amino acids, which we named Gcr1^U^. Therefore, cells mainly produce two Gcr1 isoforms, Gcr1^U^ and Gcr1^S^.

When the *GCR1* gene was first cloned, an open reading frame (ORF) of *GCR1* corresponding to that which generates Gcr1^U^ was predicted without recognizing the presence of an intron (17). Later however, *GCR1* cDNA generating Gcr1^A^ was cloned from a cDNA library and mainly used in the most recent studies (18). Because the generation of two Gcr1 forms was only recently identified, studies on Gcr1 were conducted with either Gcr1^U^ or Gcr1^A^ without considering both isoforms. Although the spliced mRNA generating Gcr1^A^ was categorized as a minor isoform (15,16), it was initially annotated as a spliced form (18) and was used in several previous studies on Gcr1. The amino acid sequences of Gcr1^A^ and Gcr1^S^ are almost identical except for a few N-terminal amino acids, as VCT from position 2 to 4 of Gcr1^A^ is replaced by QTSVDST in Gcr1^S^ (15). Considering that these N-terminal amino acids are not critical for known Gcr1 function, Gcr1^A^ and Gcr1^S^ may have similar characteristics. In a previous study based on expression of the two isoforms, Gcr1^U^ and Gcr1^S^, from episomal plasmids in *GCR1* deletion mutant, cells showed normal growth only when both Gcr1 isoforms were expressed, suggesting that each isoform might have a complementary role to support cell growth (16). However, it is not yet elucidated whether these two isoforms play different roles.

In this study, to understand the differential functions of the Gcr1^U^ and Gcr1^S^ isoforms, we generated *S. cerevisiae* strains expressing either Gcr1^U^ or Gcr1^S^ by using CRISPR/Cas9-mediated genome editing, which allows a minimum of genomic perturbation and native-level expression of the genes. Here, we show that either Gcr1^U^ or Gcr1^S^ can support normal cell growth by regulating largely the same target genes. However, Gcr1^S^ works mainly as a dimer without Gcr2, whereas Gcr1^U^ works as a monomer forming a heterocomplex with the Gcr2 dimer, revealing that previously suggested two working models of Gcr1 are mediated by each isoform of Gcr1.

## MATERIALS AND METHODS

### Yeast strains and culture conditions

*Saccharomyces cerevisiae* strains used in this study are listed in Supplementary Table S1. All strains were derived from *S. cerevisiae* BY4741 (*MATa his3Δ1 leu2Δ0 met15Δ0 ura3Δ0*). Cells were grown in YPD medium (1% yeast extract, 2% bacto-peptone, and 2% dextrose) or in synthetic complete (SC) medium (0.67% yeast nitrogen base without amino acids, 2% glucose, and 0.2% amino acids dropout mixture suitable for plasmid selection). Cell growth was detected by measuring the optical density (OD) at 600 nm with spectrophotometer (Varian Cary 50 UV-vis, Agilent Technologies, USA). OD_600_ of 0.5 pre-cultured cells were cultivated in 10 mL medium in a 50 mL Erlenmeyer flask at 30°C with shaking at 170 rpm.

### Construction of plasmids and yeast strains

Genome editing using CRISPR/Cas9 system was performed as previously described (19). Briefly, Coex413-Cas9 vector containing proper gRNA and donor DNA were introduced into *S. cerevisiae* and selected on SC-His medium. The genome-edited strains were confirmed by both PCR and sequencing. The gRNA and primer sequences for donor DNA are listed in Supplementary Table S3 and S4.

Plasmids used in this study are listed in Supplementary Table S2. To produce JHY9100 and JHY9200 strains, expressing only Gcr1^U^ and Gcr1^S^, respectively, plasmids containing each *GCR1* form were generated first. DNA fragment containing the *GCR1* ORF flanked by 500-bp upstream (P*_GCR1_*) and 500-bp downstream (T*_GCR1_*) regions was PCR-amplified and cloned between *Spe*I and *Xho*I sites of pRS413, generating p413Gcr1^WT^. Next, p413Gcr1^U^ and p413Gcr1^S^ harboring *GCR1*(Δ1–574) and *GCR1*(Δ4–742) were generated by site directed mutagenesis of p413Gcr1^WT^ and used as PCR templates to produce donor DNAs to generate JHY9100 and JHY9200 strains using the CRISPR/Cas9 system.

JHY9210, 9211 and 9212 strains were generated by integrating expression cassettes for *GCR1^U^* (1-168), *GCR1^U^* (1-168)^T34C^ and *GCR1^U^* (1-168)^T149C^, respectively, at *ura3Δ0* site in the genome of JHY9200 using Coex416-Cas9-gURA3Δ0 plasmid. To generate the expression cassettes, P*_GCR1_* and T*_GCR1_* were cloned into pRS416 vector using *Sac*I/*Xba*I and *Xho*I/*Kpn*I sites, respectively, resulting in p416GCR1PT. Next, p416GCR1-USS was generated by cloning *GCR1^U^* (1–168) between the *Xba*I and *Xho*I sites of p416GCR1PT. p416GCR1-USS^F12L^ and p416GCR1-USS^L50P^ containing the expression cassettes for *GCR1^U^* (1-168)^T34C^, and *GCR1^U^* (1-168)^T149C^, respectively, were generated by site directed mutagenesis of p416GCR1-USS. Donor DNA containing the expression cassette flanked by 35-bp homology regions targeting the *ura3Δ0* site was amplified by PCR and transformed into JHY9200. Donor DNAs for other strains were produced by PCR without template DNA using primers containing 35-bp homology regions targeting the integration site and 20-bp overlapped base pairs between two primers. To overexpress *ALD4* gene, PCR-amplified *ALD4* ORF was cloned into p416TEF between *Spe*I and *Sal*I sites, generating p416TEF-ALD4. PCR-amplified expression cassette containing P_TEF1_–*ALD4*–T_CYC1_ was transformed with Coex416-Cas9-gURA3Δ0 resulting in JHY9202A strain.

JHY9310 and JHY9320 containing *GCR1^U^-TAP* and *GCR1^S^-TAP*, respectively, were generated by homologous recombination by introducing DNA fragment PCR amplified from *S. cerevisiae GCR1*-TAP strain (20) into JHY9100 and JHY9200, respectively. Strains with 5Flag-tagged *GCR1* (JHY9302, 9312 and 9322), *GCR2* (JHY9311 and 9321) and USS domain (JHY9210F, 9211F and 9212F) were generated by using DNA fragments amplified from pFA6a-5Flag-hphMX6 vector as a template. JHY9322U1, 9322U2 and 9322U3 strain was generated by integrating the USS expression cassette into JHY9322 strain, as described for the production of JHY9210∼9212 strains.

### Screening suppressor mutants of JHY9105

To select *GCR1*-USS mutants, which can suppress the growth defect of JHY9105 (*GCR1^U^gcr2Δ*), USS mutant library was generated by error-prone PCR of *GCR1^U^* (1–168) DNA fragment. Error-prone PCR was conducted with 5 mM of MgCl_2_ or each 1 mM of dCTP/dTTP, using Taq polymerase (BioFACT^TM^, Biofact, Korea). The mutant library was introduced into JHY9105 as donor DNA with Coex413-Cas9-gGCR1USS expressing a gRNA targeting the *GCR1*-USS locus. The transformants were spread on SC-His medium and suppressor mutants were selected based on the bigger colony size, and the mutated sequences were analyzed by DNA sequencing of the USS domain.

### mRNA extraction and cDNA synthesis

Total RNA was extracted from yeast cells using the hot phenol method (21). For cDNA synthesis, 1 μg of heat-denatured total RNA was mixed with total 30 μL reaction mixture (containing 4 μL oligo dT, 2 μL M-MLV reverse transcriptase, and 4 μL each of 10 mM dNTPs) and incubated at 42°C for 60 min, and then reverse transcription reaction was terminated by heating at 75°C for 15 min.

### Quantitative reverse transcription PCR (qRT-PCR) analysis

The relative amount of target mRNA was determined by qRT-PCR of the synthesized cDNA. 5 μL of cDNA was amplified by SYBR Green I master mix (Roche Life Science, Germany) and gene-specific primers with 45 cycles of 95°C for 20 s, 60°C for 20 s and 72°C for 20 s using a Lightcycler 480 II system (Roche Life Science, Germany). The crossing point (Cp) values were processed using Light Cycler 480 software version 1.5. Expression levels of target genes were normalized by selected reference gene, *TFC1*. Primers used in qRT-PCR was listed in Supplementary Table S5.

### RNA-seq and analysis

RNA-seq libraries were prepared using a TruSeq Standard mRNA Sample Prep Kit (Illumina) according to the manufacturer's protocol. Paired-end sequencing with 100 cycles was performed using a HiSeq2500 (Illumina) instrument according to manufacturer's protocol. The quality of raw reads was assessed with FastQC (version 0.11.9); the quality scores were >Q30, which indicated high quality. Clean reads with high quality scores were processed using the Tuxedo protocol (22) with TopHat2 (version 2.1.1) (23) and Cufflinks (24). Reads for each sample were aligned to the yeast reference genome (sacCer3 assembly) using TopHat2. Gene expression quantification was performed using Cufflinks, and fragments per kilobase of transcript per million reads mapped (FPKM) was calculated as the expression value. Differential expression analysis between exponential phase and diauxic shift of Gcr1^U^ and Gcr1^S^ with two replicates were performed using Cuffdiff (24), with the cut-off set at *P* < 0.01 and ≥1.5-fold change. Their expression pattern of targeted genes of Gcr1^U^ and Gcr1^S^ was visualized as heatmap by using MeV (http://mev.tm4.org). Expressions of genes were shown as Z-score for FPKM.

### *In vivo* TAP pull-down assay and immunoblotting

Cells were grown until half of the glucose was consumed and lysed in lysis buffer [50 mM Tris–HCl (pH 7.5), 150 mM NaCl, 2 mM MgCl_2_, 0.1% NP40] supplemented with 0.1% protease inhibitor cocktail (Calbiochem, USA) and 1 mM PMSF using acid-washed glass bead. After repeating 30 s on/90 s off cycle for 10 times, cell debris were centrifuged down for 20 min and total protein concentration of the supernatant was analyzed by Bradford assay. 800 μg of proteins were used for TAP pull down with 20 μL of IgG Sepharose 6 Fast Flow resin (GE healthcare, USA) for 1 h, washed three times with lysis buffer, and eluted by boiling with 5× sample buffer. Samples were resolved by size on 6% SDS-PAGE gel and analyzed by western blotting with anti-DDDDK antibody (MBL life science, USA) for flag tag and anti-mouse IgG antibody (Sigma-Aldrich, USA) for TAP tag. Blotted membrane was treated with proper HRP-conjugated secondary antibody and visualized by G::box Chemi-XL (Syngene, USA).

### Chromatin immunoprecipitation (ChIP)

Cells were cross-linked with final 1% of formaldehyde for 25 min followed by 5 min quenching with 250 mM glycine. Harvested cells were washed with ice-cold TBS [50 mM Tris–HCl (pH 7.4), 150 mM NaCl] three times and ChIP-lysis buffer [50 mM HEPES-KOH (pH 7.5), 150 mM NaCl, 1 mM EDTA, 1% Triton X-100, 0.1% Sodium deoxycholate, 0.2% SDS] once. Cells were lysed in 200 μL lysis buffer supplemented with 0.1% protease inhibitor cocktail (Calbiochem, USA) and 1 mM PMSF using acid-washed glass beads and periodical vortexing. 800 μL of lysis buffer was added to the lysates and sonicated for 20 s using sonicator (Vibra-cell, Sonics & materials inc., USA) with amplitude 22%, 12 times for 6 h samples and 14 times for 12 h samples. Crude lysates were centrifuged for 20 min to eliminate debris. 200 μL of lysis buffer was supplemented to equal volume of sonicated lysates and incubated at 4°C for overnight with anti-DDDDK antibody followed by 1 h incubation with the Protein G Plus agarose bead (Santa Cruz Biotechnology, USA). For TAP-tagged protein, the lysates were incubated with IgG-sepharose beads (GE healthcare, Sweden) at 4°C for 1 h. Beads were washed with lysis buffer without SDS, twice in high salt lysis buffer [50 mM HEPES–KOH (pH 7.5), 500 mM NaCl, 1mM EDTA, 1% Triton X-100, 0.1% sodium deoxycholate], once with LiCl wash buffer [10 mM Tris–HCl (pH 8.0), 250 mM LiCl, 1 mM EDTA, 0.5% NP-40, 0.5% sodium deoxycholate], twice with TE buffer [10 mM Tris–HCl (pH 8.0), 1 mM EDTA]. DNA was eluted from beads by incubating with elution buffer [1% SDS, 250 mM NaCl] for 30 min, 65°C. Eluent was transferred into fresh tube and treated with RNase for 1 h, and Proteinase K for 2 h. After reversal of crosslinking by overnight incubation at 65°C with 100 mM NaCl, DNA was purified with DNA purification kit (Qiagen). Input samples were prepared with the same procedure except for the beads-binding and elution steps.

### ChIP-qPCR

Fold enrichment of DNA binding Gcr1-TAP or Gcr2–5Flag was determined by qPCR using Roche LightCycle 480 II. Concentrations of each target promoter DNA fragment in immuoprecipitated samples were divided by input samples first and normalized by *ACT1* promoter. Primers used in ChIP-qPCR were listed in Supplementary Table S5.

### ChIP-seq and data analysis

For ChIP-seq sample preparation, triplicate cultured cells were harvested and sonicated each and mixed up to 3 mL of total volume. For each protein tag, two sets of 600 μL samples from the mixture were incubated with proper antibody and bead followed by reverse crosslinking. Reverse crosslinked mixtures were treated with phenol–choloform–isoamylalcohol (25:24:1) and precipitated by ethanol and glycogen at –80°C. Two sets of dried pellets were dissolved in water and combined for high DNA concentration and used for analysis.

The sequencing libraries were prepared from ChIP DNA fragments (1–5 ng) of Gcr1^U^ and Gcr1^S^ with two replicates using ThruPLEX DNA-Seq Kit (TaKaRa) according to the manufacturer's protocol. In brief, DNA fragments were subjected to steps of end-repair, 3′A-tailing, and adapter ligation. Then DNA was PCR amplified (15 cycles) and purified. Single-end sequencing with 50 cycles was performed using a HiSeq2500 (Illumina) instrument according to manufacturer's protocol.

Read quality was assessed using FastQC (v0.10.1) (25), showing about 90% bases above Q30 across all samples. Reads were aligned to the yeast reference genome (sacCer3 assembly) using BWA (v0.7.15) (26) with the allowance of two mismatches, and redundant reads with identical coordinates were filtered out using Picard (v2.92) (https://broadinstitute.github.io/picard/index.html). ChIP peaks was called using HOMER (27) ‘findPeaks’ with –style ‘factor’. Called peaks were filtered with the following conditions: (i) peak score ≥100, (ii) poisson *P*-value threshold relative to local tag count <1*E*–10, (iii) fold enrichment over local tag count ≥2 and (iv) relation to genes. Peaks overlapped between exponential phase and diauxic shift of Gcr1^U^ and Gcr1^S^ were further selected to compare their binding pattern in the two mutants, and ChIP reads fallen in peaks were then collected using BEDTools ‘intersectbed’ (28). Moreover, peaks that are differentially enriched between the two experiments were examined by using HOMER ‘getDifferentialPeaks.’ The resulting peak calls were annotated using HOMER annotatePeaks with a pre-configured genome annotation provided from HOMER. The enrichments of Gcr1^U^ and Gcr1^S^-bound regions in the yeast genome were plotted using seqMiner (29) with ChIP reads fallen in peaks.

### HPLC analysis

To determine the concentrations of metabolites, 600 μL of culture supernatants were filtered through a 0.22 μm syringe filter and analyzed by high performance liquid chromatography (HPLC) with BioRad Aminex HPX-87H column. 5 mM H_2_SO_4_ was used as a mobile phase at a flow rate 0.6 mL/min and column and refractive index (RI) detector temperature were maintained at 60°C and 35°C, respectively.

## RESULTS

### No phenotypic difference was observed between strains producing only Gcr1^U^ or Gcr1^S^

**Figure 1.**
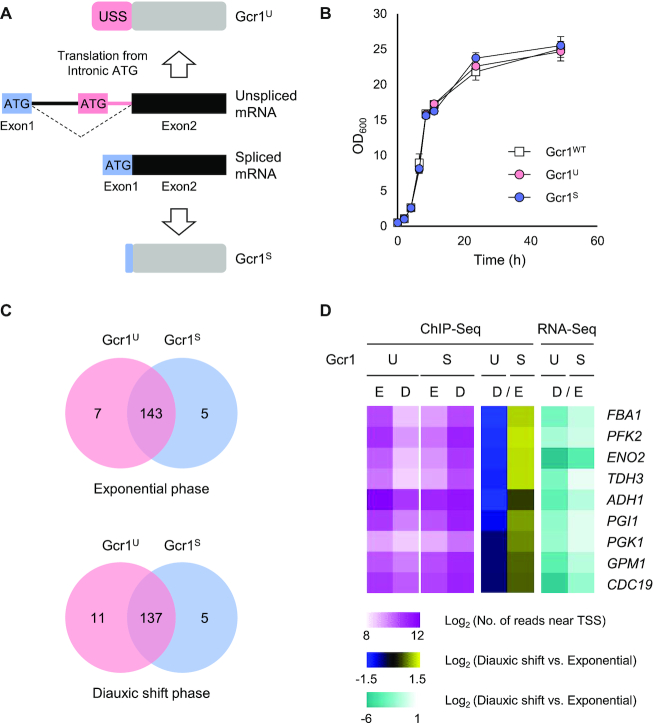
Gcr1^U^ and Gcr1^S^ mostly bind to the same targets, but with different intensities depending on growth phase. (**A**) Schematic view of production of Gcr1^U^ and Gcr1^S^ from un-spliced and spliced forms of mRNA, respectively. Without splicing, translation starts from the intronic start codon (pink box) and generates Gcr1^U^. When splicing occurs using the major 5′ and 3′ splicing sites, the start codon in the exon 1 (blue box) is used to generate Gcr1^S^. USS indicates un-spliced form-specific domain. (**B**) Growth curves of the Gcr1^WT^, Gcr1^U^ and Gcr1^S^ strains. Cells were grown in YPD media containing 2% glucose. Error bars indicate the standard deviations of three independent experiments. (**C**) Venn diagrams show the number of overlapping target genes of Gcr1^U^ and Gcr1^S^ identified by ChIP-seq analyses at exponential and diauxic shift phases. The binding pattern of target genes are visualized by heatmap in Supplementary Figure S3 and listed in Supplementary Table S6. Genes used in the Venn diagram are listed in Supplementary Table S7. (**D**) Relative abundance of ChIP-seq ‘reads’ related to TSS of nine targeted genes at exponential (E) and diauxic shift (D) phases were visualized for Gcr1^U^ (U) and Gcr1^S^ (S) strains. The log_2_ fold-changes (D versus E) of ‘reads’ abundance were visualized for each Gcr1 isoform. Target gene expression levels were examined by RNA-seq and log_2_ fold-changes (D versus E) of FPKM were visualized as heat maps for each Gcr1 isoform. The color scales and the names of the nine target genes are indicated.

To generate strains producing only Gcr1^U^ or Gcr1^S^ in a native state, CRISPR/Cas9-mediated genome editing was conducted in the *S. cerevisiae* BY4741 strain. The Gcr1^U^ strain, producing only the un-spliced form of the Gcr1 protein, was generated by deleting an exon 1 (a start codon) and a 574-bp intron region upstream of the intronic start codon of the *GCR1* gene (Figure 1A). The Gcr1^S^ strain, which creates only the spliced form of Gcr1 protein, was generated by deleting a 739-bp intron region between the major 5′ and 3′ splice sites (Figure 1A). Compared with the Gcr1^S^ protein, the Gcr1^U^ protein has an additional 55 amino acids at the N-terminus, which was named the USS (un-spliced form specific) domain (Figure 1A).

In a previous study using plasmid-based gene expression, cells expressing Gcr1^U^ or Gcr1^S^ alone showed growth defects compared with cells expressing both isoforms (16). Therefore, we first examined growth rates of our strains in rich YPD medium containing 2% glucose. However, unlike the previous study, both strains showed the same growth rates as the wild-type BY4741 strain (Figure 1B). We also assessed different culture conditions including SC minimal media, discrete concentrations of glucose, a variety of carbon sources, and various environmental stress conditions, but the Gcr1^U^ and Gcr1^S^ strains showed no significant differences in growth compared with wild type (Supplementary Figure S1).

To confirm the expression of the specific Gcr1 isoform in each strain, we also generated strains expressing Gcr1^U^ or Gcr1^S^ tagged with TAP at the C-terminus. Gcr1^U^-TAP and Gcr1^S^-TAP strains also showed no growth defect as compared with wild-type Gcr1-TAP strain (Supplementary Figure S2A). In Western blotting analysis, wild-type Gcr1-TAP strains showed two Gcr1 protein bands corresponding to Gcr1^U^ and Gcr1^S^ (Supplementary Figure S2B). However, the Gcr1^U^-TAP and Gcr1^S^-TAP strains showed only its respective isoform. Production of different isoforms in each strain was also confirmed by tagging Gcr1 with 5Flag (Supplementary Figure S2C). These data indicate that the normal growth phenotypes of the Gcr1^U^ and Gcr1^S^ strains are not due to the concurrent production of two Gcr1 isoforms.

### Gcr1^U^ and Gcr1^S^ mostly bind to the same targets, but with different intensities depending on growth phase

Normal growth of Gcr1^U^ and Gcr1^S^ strains suggest that the functions of Gcr1^U^ and Gcr1^S^ are largely indistinguishable from each other, at least under our laboratory culture conditions. Therefore, we next investigated whether Gcr1^U^ and Gcr1^S^ regulate any other sets of target genes. Genome-wide binding targets of Gcr1^U^ and Gcr1^S^ were examined at exponential and post-diauxic shift phases by ChIP-seq analysis. We identified 155 genes showing ‘reads’ enrichment around the transcription start sites (TSS) (Supplementary Figure S3, Supplementary Table S6). Most of the identified genes overlapped between Gcr1^U^ and Gcr1^S^ during both growth phases (Figure 1C, Supplementary Table S7). In agreement with previous studies showing Gcr1-dependent regulation of transposable elements (Ty) (30–32), 33 peaks were located near transposable elements. After filtering out the Ty elements, 26 tRNA genes, seven genes near telomeres, and 50 unnamed genes, the 39 remaining binding targets of Gcr1^U^ and Gcr1^S^ mainly represented glycolytic genes consistent with the known role of Gcr1 (Supplementary Table S8).

Although we could not identify any meaningful target genes specific for either Gcr1^U^ or Gcr1^S^, the DNA binding for each Gcr1 isoform showed a different tendency depending on growth phase. ChIP-seq analysis of Gcr1^U^ showed a decrease in the ‘reads’ abundance of the targets from the exponential to the diauxic shift phases, whereas ChIP-seq analysis of Gcr1^S^ showed an opposite trend (Figure 1D). We also examined expression levels of the target genes in Gcr1^U^ and Gcr1^S^ strains by using RNA-seq analysis. Expression of the glycolytic genes decreased after a diauxic shift in both Gcr1^U^ and Gcr1^S^ strains, reflecting the contribution of glycolysis in the presence of glucose. However, the fold-changes in target gene expression (diauxic shift/exponential) were greater in the Gcr1^U^ strain than in the Gcr1^S^ strain (Figure 1D). Therefore, Gcr1^U^ may work mainly during the exponential phase, but increased Gcr1^S^ binding to the target gene promoters after diauxic shift might support residual expression of glycolytic genes even after glucose depletion. Taken together, Gcr1^S^ and Gcr1^U^ might play differential roles not by regulating different sets of target genes, but by differential binding to the same genes depending on growth conditions.

### Deletion of specific Gcr1 domains or *GCR2* revealed the phenotypic differences between Gcr1^U^ and Gcr1^S^ strains

**Figure 2.**
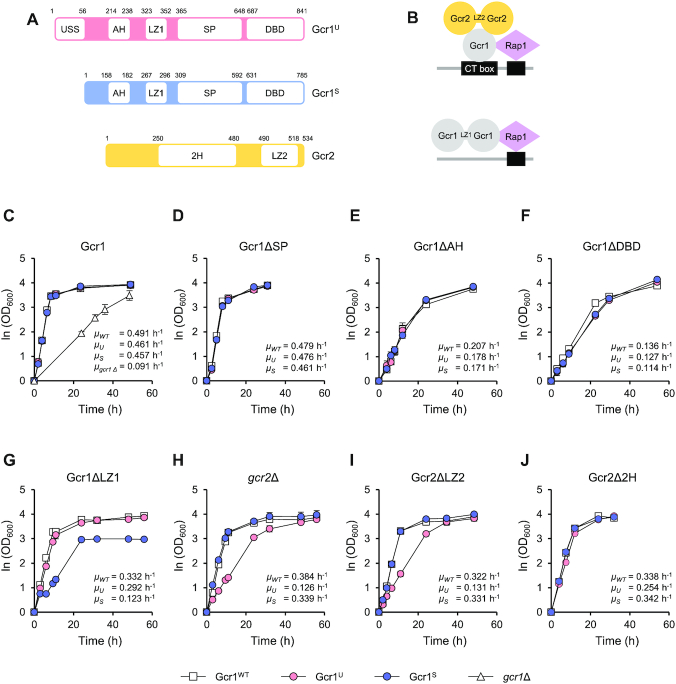
Deletion of specific domains of Gcr1 and Gcr2 revealed the phenotypic differences between the Gcr1^U^ and Gcr1^S^ strains. (**A**) Functional domains of Gcr1 and Gcr2. AH, alpha helix; LZ1, leucine zipper of Gcr1; SP, serine-proline rich; DBD, DNA binding domain; 2H, Gcr2 region homologous to Gcr1; LZ2, leucine zipper of Gcr2. (**B**) Previously suggested working models of Gcr1 and Gcr2 (12). It is hypothesized that a Gcr1 (monomer or dimer) associates with a Gcr2 dimer to activate glycolytic genes, whereas the Gcr1 homodimer activates RP genes through interaction with Rap1. (**C**–**J**) Growth curves and specific growth rates of the Gcr1^WT^, Gcr1^U^ and Gcr1^S^ strains, and strains with the indicated mutations on three different Gcr1 backgrounds. Cells were grown in YPD media containing 2% glucose. The results of three (**C**, **G**, **H**, **I**) or two (**D**, **E**, **F**, **J**) independent experiments were averaged and plotted with standard deviations.

To test the hypothesis that the regulatory mechanisms of Gcr1^U^ and Gcr1^S^ were different, we deleted previously identified domains of Gcr1 including the alpha helix (AH), leucine zipper (LZ1), serine-proline rich (SP), and DNA binding domains (DBD) (11,12) using CRISPR/Cas9-mediated genome editing, which allowed seamless editing of each domain into the genome (Figure 2A). In addition, *GCR2*, which encodes a coactivator of Gcr1 or its 2H or LZ domain (LZ2) was deleted in cells expressing Gcr1^WT^, Gcr1^U^ or Gcr1^S^, respectively (Figure 2A).

As previously reported (13), the *GCR1* deletion strain showed a severe growth defect in YPD media (Figure 2C). However, deletion of the SP domain did not affect growth rates against all three Gcr1 backgrounds (Figure 2D). On the other hand, deletion of the AH domain and DBD led to reduced growth rates in all three Gcr1 backgrounds, suggesting that these domains affect Gcr1 activity irrespective of the isoforms (Figure 2E and F). In agreement with previous data showing that Gcr1 lacking a DBD can support activation of glycolytic genes through interaction with Rap1 (9) (Figure 2B), the Gcr1ΔDBD strains showed slightly higher specific growth rates than the *gcr1Δ* strain. However, deletion of the LZ1 domain, which plays an important role in Gcr1 homodimerization (12), showed differential effects depending on the Gcr1 isoform. LZ1 deletion led to a significant reduction in cell growth rate in the Gcr1^S^ strain, but not in the Gcr1^WT^ and Gcr1^U^ strains (Figure 2G). Moreover, the Gcr1^S^ΔLZ1 strain showed a lower final cell density than the other strains (Figure 2G). Therefore, homodimerization may be essential for the activity of Gcr1^S^, but not for Gcr1^U^.

On the contrary, *GCR2* deletion mainly affected Gcr1^U^, but not Gcr1^S^ and Gcr1^WT^ (Figure 2H). The Gcr1^U^*gcr2*Δ strain showed a lower growth rate than the other two strains, but final cell density was not affected by *GCR2* deletion (Figure 2H). Deletion of the LZ domain of Gcr2 (LZ2), which is involved in Gcr2 homodimerization, exhibited the same effects as *GCR2* deletion, resulting in a severe growth defect only in the Gcr1^U^ background strain (Figure 2I). On the other hand, deletion of the 2H domain of Gcr2 led to mild growth defects in all strains (Figure 2J). These results indicate that Gcr1^U^ acts as a transcription factor with the help of Gcr2 homodimer, but Gcr1^S^ can be functional without Gcr2.

**Figure 3.**
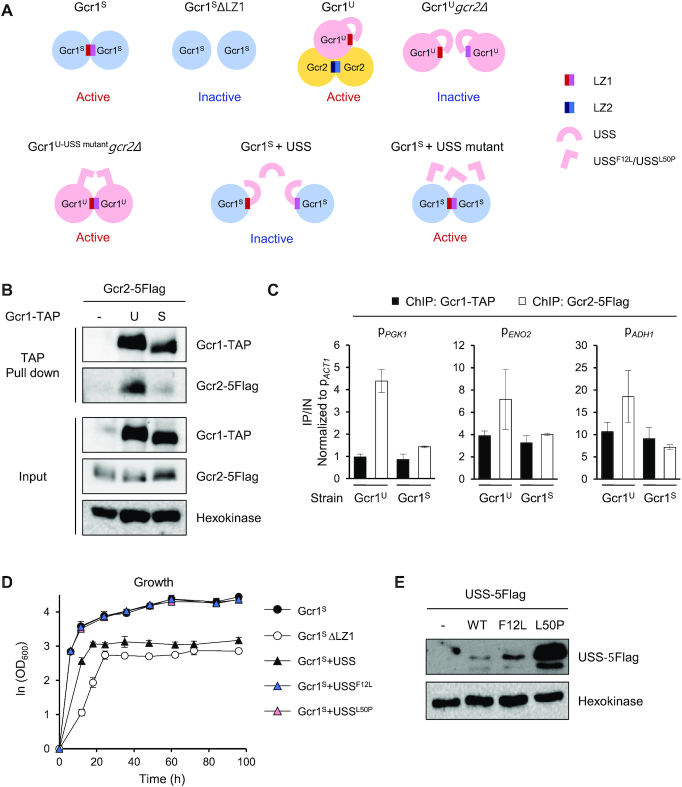
Working models of Gcr1^U^ and Gcr1^S^. (**A**) Working models of Gcr1^U^, Gcr1^S^ and the USS domain. Gcr1^S^ mainly works as a homodimer linked through the LZ1 domain and is thereby inactivated by deletion of the LZ1 domain (Gcr1^S^ΔLZ1). In contrast, Gcr1^U^ mainly acts as a monomer forming a heterocomplex with a Gcr2 dimer, and is inactivated in the absence of Gcr2 (Gcr1^U^*gcr2Δ*). The USS domain inhibits dimerization of Gcr1^U^. The mutants USS^F12L^ and USS^L50P^ may suppress Gcr1^U^*gcr2Δ* by allowing dimerization of Gcr1^U^. The USS domain, but not the USS mutants, inhibits dimerization Gcr1^S^ in a trans-acting manner, thus inactivating Gcr1^S^. (**B**) Stronger Gcr2 binding to Gcr1^U^ than to Gcr1^S^. The interaction between Gcr1 and Gcr2 was detected by *in vivo* TAP pull-down assay using strains co-expressing Gcr1^U or S^-TAP and Gcr2–5Flag. Gcr1-TAP and Gcr2–5Flag were detected by immunoblotting with IgG and anti-Flag antibody, respectively. Hexokinase was used as a loading control. (**C**) Higher Gcr2 binding to the promoters of glycolytic genes in Gcr1^U^ strain than in Gcr1^S^ strain. Binding of Gcr1^U or S^-TAP and Gcr2–5Flag to the indicated target promoters were detected by ChIP experiments using strains co-expressing Gcr1^U^-TAP and Gcr2–5Flag (Gcr1^U^) or Gcr1^S^-TAP and Gcr2–5Flag (Gcr1^S^) and are indicated as fold enrichments normalized to the *ACT1* promoter. Each value represents the average ± standard deviations from two independent experiments. (**D**) Growth curves of the Gcr1^S^ strains expressing the USS domain or its suppressor mutant F12L or L50P in comparison with Gcr1^S^ and Gcr1^S^ΔLZ1 strains (*n* = 3, average ± standard deviations). (**E**) Expression levels of the USS domains. Cell lysates of the Gcr1^S^ strain expressing wild-type or mutant USS-5Flag domain were subjected to immunoblotting with anti-Flag antibody to detect expression levels of the USS domains. Hexokinase was used as a loading control and the Gcr1^S^ strain without the USS domain was used as a negative control.

Based upon all of these results, we hypothesized a working model that Gcr1^U^ and Gcr1^S^ might be activated through different regulatory mechanisms (Figure 3A). Gcr1^S^ activity was reduced by deleting the LZ1 domain, suggesting that Gcr1^S^ mainly works as a homodimer connected through its LZ1 domain. In contrast, Gcr1^U^ activity was reduced by deletion of the *GCR2* gene or the LZ2 domain of Gcr2, suggesting that Gcr1^U^ works as a monomer that forms a heterocomplex with the Gcr2 homodimer. To verify this working model, we investigated whether Gcr1^U^ has higher Gcr2-binding affinity than Gcr1^S^. We used strains expressing Gcr1^S^-TAP or Gcr1^U^-TAP together with Gcr2–5Flag. In agreement with our working model, the TAP-pull down experiment showed stronger co-immunoprecipitation of Gcr2 to Gcr1^U^ than to Gcr1^S^ (Figure 3B). We also investigated the binding between Gcr1 and Gcr2 by using a ChIP experiment. Because Gcr2 can bind to DNA only through interacting with Gcr1, the DNA binding intensity of Gcr2 reflects its binding affinity to Gcr1. Although Gcr1^U^ and Gcr1^S^ showed similar binding intensities to target promoters of the glycolytic genes, Gcr2 showed a higher DNA binding intensity in cells expressing Gcr1^U^ as compared with cells expressing Gcr1^S^ (Figure 3C). These experiments support the idea that Gcr1^U^ is the major binding partner of Gcr2.

### The USS domain inhibits homodimerization of the Gcr1^U^ protein

The only difference between the Gcr1^U^ and Gcr1^S^ proteins is the additional 55 N-terminal residues of Gcr1^U^, referred to as the USS domain. Gcr1^U^, which contains the USS domain, is more likely to interact with Gcr2 rather than forming a homodimer, whereas Gcr1^S^ seems to act mainly as a homodimer without Gcr2 (Figure 3A). Therefore, the USS domain could play a role in inhibiting Gcr1 dimerization while facilitating the interaction with Gcr2. Considering the fact that cell growth is little affected by deletion of *GCR2* in the Gcr1^S^ strain, the growth defect of Gcr1^U^*gcr2*Δ strain might be due to inhibition of Gcr1^U^ dimerization by the USS domain in absence of Gcr2, possibly through intramolecular interaction masking the LZ1 domain (Figure 3A). If this is the case, the USS domain might contain some residues essential for inhibiting Gcr1 dimerization. Mutation of those residues could rescue the growth defect of the Gcr1^U^*gcr2*Δ strain by allowing homodimerization of Gcr1^U^, thus activating Gcr1^U^ without Gcr2 (Figure 3A). To examine this hypothesis, we performed random mutagenesis of the USS domain in the Gcr1^U^*gcr2*Δ strain using the CRISPR/Cas9 system, and isolated two suppressor mutants (F12L and L50P) with improved growth. The USS domain with these point mutations is expected to lose its ability to inhibit Gcr1 dimerization (Figure 3A). If the USS domain acts through intramolecular interaction, the USS domain alone could play the same inhibitory role in a trans-acting manner. To test this possibility, we examined whether the USS domain could inhibit dimerization of Gcr1^S^, which would lead to a growth defect in the Gcr1^S^ strain, mimicking the Gcr1^S^ΔLZ1 strain (Figure 3A). In agreement with this hypothesis, when a DNA fragment encoding the USS domain was inserted into the *ura3Δ0* locus in the Gcr1^S^ strain and expressed under the control of the native *GCR1* promoter and terminator, the cell growth pattern was very similar to that of the Gcr1^S^ΔLZ1 strain, exhibiting a reduced growth rate and decreased final cell density (Figure 3D). However, the growth rate of the Gcr1^S^ strain was not affected when the USS^F12L^ or USS^L50P^ suppressor mutant was expressed (Figure 3D), further confirming that these mutant USS domains cannot inhibit Gcr1 dimerization (Figure 3A). The protein expression levels of USS^F12L^ and USS^L50P^ were even higher than that of the wild type USS (Figure 3E), indicating that the lack of Gcr1^S^ inhibition by these mutants was not due to defects in their expression.

### Cells expressing Gcr1^S^ΔLZ1 showed a defect in respiration

**Figure 4.**
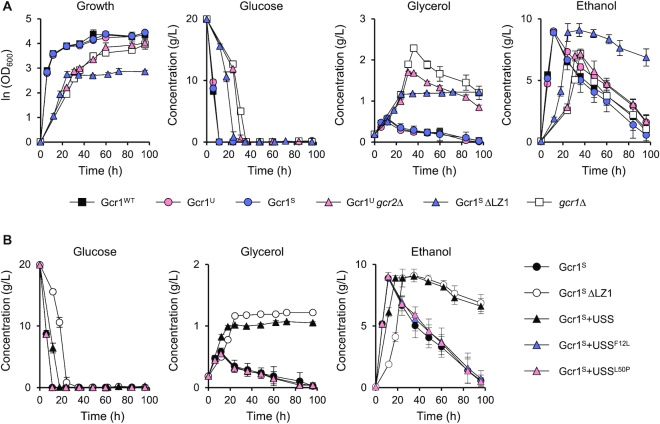
Growth curves and metabolite profiles of strains expressing various Gcr1 isoforms and mutants. (**A**) Cell growth curves and concentrations of metabolites (glucose, glycerol, and ethanol) in the medium during the cell growth in YPD medium (average ± standard deviations, *n* = 3). (**B**) Metabolite profiles of the Gcr1^S^ strains expressing the USS domain or its suppressor mutant F12L or L50P in comparison with Gcr1^S^ and Gcr1^S^ΔLZ1 strains (average ± standard deviations, *n* = 3).

Both Gcr1^S^ΔLZ1 and Gcr1^U^*gcr2Δ* strains showed reduced growth rates in the exponential growth phase (Figure 2G and H), reflecting reduced expression of glycolytic genes. However, the Gcr1^S^ΔLZ1 strain, but not the Gcr1^U^*gcr2Δ* strain, showed diminished final cell density, suggesting dissimilar carbon metabolic pathways of the two strains. Therefore, we examined metabolite profiles in strains expressing different Gcr1 forms. In agreement with their similar growth rates, the Gcr1^WT^, Gcr1^U^ and Gcr1^S^ strains showed similar patterns of glucose uptake, production of ethanol and glycerol, and utilization of ethanol and glycerol via respiration after glucose depletion (Figure 4A). Alternatively, the Gcr1^U^*gcr2Δ* and *gcr1Δ* strains with reduced growth and glucose uptake rates, showed lower ethanol production and higher glycerol production levels as compared with the wild type strain, but metabolized ethanol and glycerol normally after glucose depletion (Figure 4A). The Gcr1^S^ΔLZ1 strain also accumulated higher concentrations of glycerol than wild type (Figure 4A). However, the Gcr1^S^ΔLZ1 strain showed a defect in respiratory consumption of glycerol and ethanol after glucose depletion (Figure 4A).

We also confirmed the respiratory defect in the Gcr1^S^ΔLZ1 strain by analyzing metabolite profiles of the Gcr1^S^ strain expressing the USS domain, which mimics the growth phenotype of the Gcr1^S^ΔLZ1 strain. Similar to the Gcr1^S^ΔLZ1 strain, the Gcr1^S^ strain expressing the USS domain showed a defect in metabolizing glycerol and ethanol after the diauxic shift (Figure 4B). However, expression of the mutant USS^F12L^ or USS^L50P^ domain did not affect the respiration of the Gcr1^S^ strain, confirming that inhibition of Gcr1^S^ dimerization leads to a respiratory defect. Because such a respiratory defect was not observed in the *gcr1Δ* strain (Figure 4A), the monomer form of Gcr1^S^, mainly produced in the Gcr1^S^ΔLZ1 strain or in the Gcr1^S^ strain expressing the USS domain, might exert a dominant negative effect on the expression of genes involved in respiration after diauxic shift.

### Gcr1^S^ΔLZ1 strain showed a defect in inducing the respiratory genes after diauxic shift

**Figure 5.**
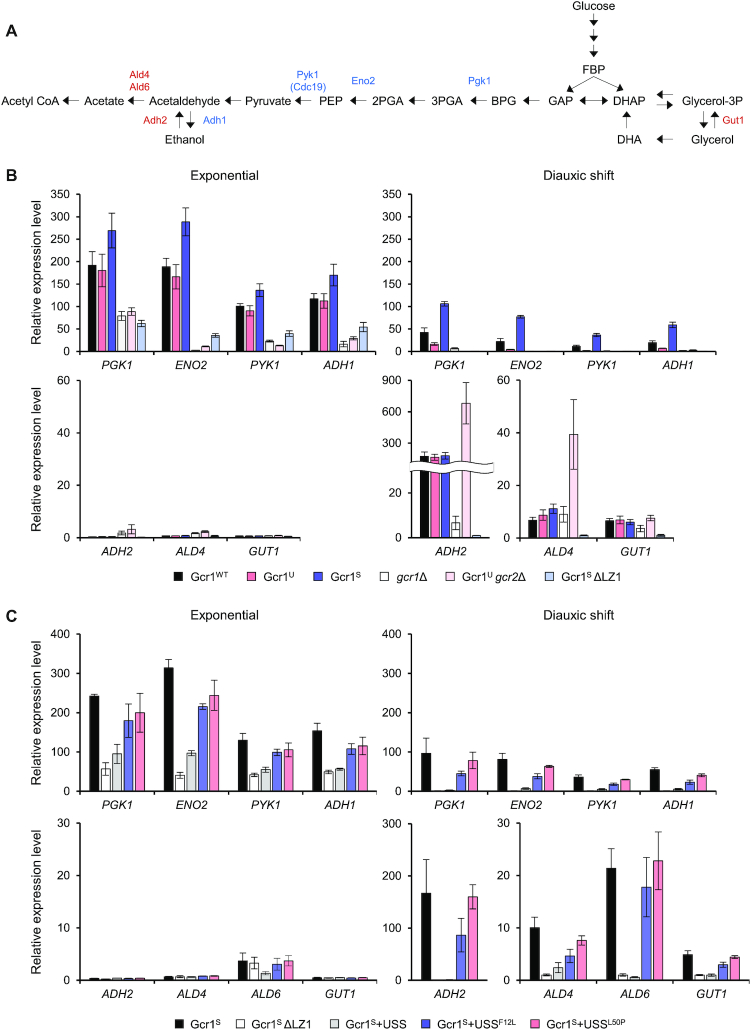
Expression of genes involved in glycolysis, ethanol production and consumption of ethanol and glycerol in strains expressing various Gcr1 isoforms and mutants. (**A**) Metabolic pathways of glycolysis, ethanol fermentation, and utilization of ethanol and glycerol. Enzymes encoded by Gcr1 target genes involved in glycolysis (Pgk1, Eno2 and Pyk1) and ethanol production (Adh1) are shown in blue. Enzymes involved in the utilization of ethanol (Adh2, Ald4, and Ald6) and glycerol (Gut1) are shown in red. (**B**) Gene expression levels in strains expressing various Gcr1 isoforms and mutants. Cells were grown in YPD media and gene expression levels at the exponential and diauxic shift phases were detected by qRT-PCR normalized to the mRNA levels of *TFC1*. Each value represents the average ± standard deviations (*n* = 3) of the relative fold-change in expression, normalized to the expression level of the Gcr1^S^ΔLZ1 strain at the diauxic shift phase. (**C**) Gene expression levels in the Gcr1^S^ strain expressing various USS domains. Cells were grown in YPD media and gene expression levels at the exponential and diauxic shift phases were detected by qRT-PCR normalized to the mRNA levels of *TFC1*. Each value represents the average ± standard deviations (*n* = 3) of the relative fold-change in expression, normalized to the expression level of the Gcr1^S^ΔLZ1 strain at the diauxic shift phase.

To confirm the effects of various Gcr1 isoforms and mutants on expression of genes involved in carbon metabolism, we investigated transcription of genes involved in glycolysis (*PGK1*, *ENO2*, *PYK1*), ethanol production (*ADH1*), ethanol utilization (*ADH2* and *ALD4*), and glycerol utilization (*GUT1*) in strains expressing different Gcr1 isoforms or mutants (Figure 5A). Gene expression levels were analyzed during the exponential and diauxic shift phases. Because of the discrete growth rates of each strain, we determined sampling time points based on the glucose concentrations remaining in the medium. Exponential growth phase samples were taken when the remaining glucose concentration was 10 g/L, and the diauxic shift phase samples were obtained when the cells consumed the entire glucose supply.

In all strains evaluated (wild type, Gcr1^U^, Gcr1^S^, *gcr1*Δ, Gcr1^U^*gcr2*Δ and Gcr1^S^ΔLZ1), expression of *PGK1, ENO2, PYK1* and *ADH1* genes, which are target genes of Gcr1, decreased upon diauxic shift. Among the wild type, Gcr1^U^ and Gcr1^S^ strains, Gcr1^S^ showed the highest expression levels of these genes throughout the growth phase (Figure 5B). Instead, the Gcr1^U^ strain exhibited the lowest expression levels of the glycolytic genes after diauxic shift (Figure 5B), suggesting that Gcr1^S^ is mainly responsible for glycolytic gene expression after glucose depletion. These results are consistent with our RNA-seq results showing greater growth-dependent fold-changes in target gene expression levels in the Gcr1^U^ strain than in the Gcr1^S^ strain (Figure 1D). However, based on the similar growth rates of the wild-type, Gcr1^U^ and Gcr1^S^ strains, such differences in glycolytic gene expression levels might not be critical for cell growth, at least under our culture conditions. As expected from their slow growth rates, the *gcr1Δ*, Gcr1^U^*gcr2Δ* and Gcr1^S^ΔLZ1 strains displayed lower expression levels of the glycolytic genes and *ADH1* throughout the growth phase.

In contrast, *ADH2*, *ALD4*, and *GUT1* genes involved in respiratory consumption of ethanol and glycerol were induced upon diauxic shift, exhibiting similar expression patterns in the Gcr1^WT^, Gcr1^U^ and Gcr1^S^ strains (Figure 5B). The Gcr1^U^*gcr2Δ* strains also showed similar induction patterns of these genes upon diauxic shift, but expression levels of *ADH2* and *ALD4* were higher than those of wild type. Such induction was not observed in the Gcr1^S^ΔLZ1 strain (Figure 5B). Therefore, the respiratory defect in the Gcr1^S^ΔLZ1 strain might be due to the failure to induce respiratory genes upon diauxic shift. The Gcr1^S^ strain expressing the USS domain, which mimics the Gcr1^S^ΔLZ1 strain in terms of cell growth and metabolite profiles, showed gene expression patterns similar to those of the Gcr1^S^ΔLZ1 strain, exhibiting defects in induction of respiratory genes as well as in expressing the glycolytic genes (Figure 5C). In agreement with the inactivity of USS^F12L^ and USS^L50P^ in preventing Gcr1 dimerization, expression of these mutant USS domains did not affect induction of respiratory genes upon diauxic shift (Figure 5C). These results further support the dominant negative role for inactive Gcr1^S^ monomer in induction of respiratory genes.

### The respiratory defect of Gcr1^S^ΔLZ1 strain could be restored by overexpressing *ALD4*

**Figure 6.**
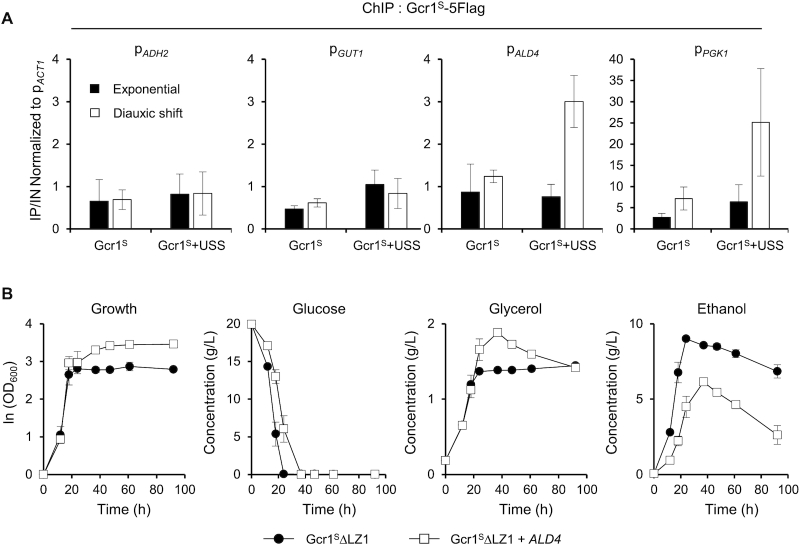
Restoration of the respiratory growth defect of Gcr1^S^ΔLZ1 strain by overexpressing *ALD4*. (**A**) Binding of the Gcr1^S^ monomer to the *ALD4* promoter after diauxic shift. Gcr1^S^ binding to the indicated promoters at the exponential and diauxic shift phases were monitored by ChIP experiments using Gcr1^S^-5Flag strains with or without USS overexpression, and indicated as fold enrichments normalized to the *ACT1* promoter. Each value represents the average ± standard deviations from three independent experiments. (**B**) Growth curves and metabolite profiles of the Gcr1^S^ΔLZ1 strain with or without overexpression of *ALD4* (*n* = 3, average ± standard deviations).

Considering the normal induction of the respiratory genes in *gcr1Δ* upon diauxic shift (Figure 5B), Gcr1 seems unnecessary for the induction of these genes. Therefore, an inactive Gcr1^S^ monomer might affect expression of respiratory genes either directly or indirectly. Respiratory genes, *ADH2*, *ALD4*, *ALD6* and *GUT1*, were not detected as Gcr1-binding targets in our ChIP-seq analysis, but other recent ChIP-exo analysis identified *ALD4* as a target where Gcr1, but not Gcr2, binds upon glucose limitation (14). Therefore, we examined whether Gcr1^S^ dimers and Gcr1^S^ monomers (Gcr1^S^ΔLZ1) could bind to respiratory gene promoters. To find any differences in DNA binding activities between Gcr1^S^ and Gcr1^S^ΔLZ1 by using ChIP experiments, we created strains expressing Gcr1^S^-5Flag or Gcr1^S^ΔLZ1–5Flag, but the Gcr1^S^ΔLZ1–5Flag strain showed a different growth phenotype compared with Gcr1^S^ΔLZ1 strain, which might be due to perturbation of protein function by the tag itself. Therefore, we instead expressed the USS domain in a Gcr1^S^-5Flag strain to mimic the phenotype of Gcr1^S^ΔLZ1 strain. In agreement with our ChIP-seq experiment, no consequential binding of Gcr1^S^ was detected to the *ADH2*, *GUT1* and *ALD4* promoters (Figure 6A). Gcr1^S^ co-expressed with the USS domain also did not bind to the *ADH2* and *GUT1* promoters, suggesting that the Gcr1^S^ monomer might indirectly affect the expression of these genes. However, when the USS domain was co-expressed with Gcr1^S^, binding to the *ALD4* promoter was detected at the diauxic shift phase, but not at the exponential phase (Figure 6A). Therefore, in accord with the previous study (14), Gcr1^S^ might bind to the *ALD4* promoter upon glucose limitation. Increased DNA binding of the Gcr1^S^ monomer than the Gcr1^S^ dimer seems to negatively affect the transcriptional induction of the *ALD4* gene upon diauxic shift, possibly by inhibiting the binding of other transcription factors. Furthermore, Gcr1^S^ co-expressed with the USS domain also showed enhanced binding to the *PGK1* promoter, suggesting that the Gcr1^S^ monomer has a higher DNA binding intensity than the Gcr1^S^ dimer in general. Consistent with the results of our ChIP-seq experiments (Figure 1D), Gcr1^S^ binding to the *PGK1* promoter increased from the exponential to the diauxic shift phase (Figure 6A), although the *PGK1* transcription diminished after diauxic shift (Figure 5B and C). Considering the reduced expression of glycolytic genes after diauxic shift even in the *gcr1Δ* strain (Figure 5B), other transcription regulators might also affect glycolytic gene expression after glucose depletion.

Our ChIP experiment suggests that *ALD4* is a direct target affected by Gcr1^S^ monomer. Because *ALD4* encodes a mitochondrial aldehyde dehydrogenase required for ethanol utilization, we examined whether *ALD4* overexpression could rescue the respiratory defect of the Gcr1^S^ΔLZ1 strain. When *ALD4* was overexpressed using the *TEF1* promoter in the Gcr1^S^ΔLZ1 strain, ethanol utilization was recovered, resulting in a higher final cell density (Figure 6B). Although Ald4 is only involved in ethanol degradation, glycerol utilization also recovered, suggesting that overexpression of *ALD4* alone can trigger cellular metabolic reprogramming to respiratory growth.

## DISCUSSION

### Gcr1^U^ and Gcr1^S^ work as different protein complexes

In this study, we investigated the differential roles of Gcr1^U^ and Gcr1^S^ by generating strains that produced only one isoform of Gcr1. The Gcr1^U^ and Gcr1^S^ strains and the other strains producing Gcr1 or Gcr2 mutants were generated by CRISPR/Cas9-mediated genome editing with minimal genomic perturbations. In a previous study, when each isoform alone was expressed from a CEN-based low-copy number plasmid in the *GCR1* deletion strain, cells exhibited growth defects as compared with cells expressing both isoforms (16). Unexpectedly however, our strain producing only Gcr1^U^ or Gcr1^S^ did not show any noticeable growth defects under normal and various stress conditions. Such dissimilar results might be mainly due to the difference in *GCR1* expression levels depending on the experimental designs. Because Gcr1 is one of the key transcription factors controlling cell growth, even slight perturbation of its expression level might affect cell growth. In fact, we observed some changes in cell growth rates when Gcr1^U^ or Gcr1^S^ was expressed from a plasmid vector (data not shown).

Although strains producing only Gcr1^U^ or Gcr1^S^ did not show any growth defects, we identified disparate working models for Gcr1^U^ and Gcr1^S^ by investigating the deletion of various Gcr1 functional domains and Gcr2 and their effects. Our genetic and biochemical evidence suggests that Gcr1^U^ mainly works as a monomer forming a heterocomplex with a Gcr2 dimer, whereas Gcr1^S^ works as a homodimer without Gcr2 binding. The N-terminal 55-amino acid USS domain, which exists only in Gcr1^U^, inhibited Gcr1 homodimerization, playing a key role in determining the formation of distinctive Gcr1 complexes. The USS domain even inhibited Gcr1^S^ dimerization in a trans-acting manner, suggesting that intramolecular interaction of the USS domain might prevent LZ1-dependent dimerization of Gcr1^U^. Cells expressing Gcr1^U^ΔLZ1 showed a slightly reduced growth rate, indicating that Gcr1^U^ monomer interacts with Gcr2 to form an active complex. However, cells expressing Gcr1^S^ΔLZ1 displayed a severe growth defect, suggesting that the Gcr1^S^ monomer cannot form an active heterocomplex with Gcr2. Although pull-down and ChIP experiments revealed that Gcr1^S^ has weaker Gcr2 binding activity than Gcr1^U^, marked binding of Gcr2 to the target promoters was still observed in the Gcr1^S^ strain, suggesting that the USS domain is not absolutely necessary for Gcr2 binding. Therefore, the USS domain might contribute to Gcr2-dependent activation of Gcr1^U^. In this case, Gcr2 binding to the Gcr1^S^ monomer might not be enough to induce the proper conformational changes for Gcr1^S^ activation.

The presence of two forms of the Gcr1 complex has been suggested in a previous study carried out with Gcr1^A^ (10,12), in that the Gcr1 homodimer and Gcr1-Gcr2 heterocomplex may be involved in the regulation of the ribosomal protein (RP) genes and glycolytic genes, respectively (12). Although it is controversial whether Gcr1 regulates RP genes, now it is apparent that the effects of Gcr1 on RP gene expression are indirect, and correlate with cell growth. Recent ChIP-exo (14) experiments, as well as our ChIP-seq analyses, revealed that the RP genes are not direct targets of Gcr1. Assuming that Gcr1^A^ mainly works as a homodimer like Gcr1^S^, cells expressing the dimerization-defective Gcr1^A^ would have a growth defect concomitant with reduced expression of RP genes, which in turn might lead to misinterpretation that the Gcr1 homodimer is required for RP gene transcription.

### Biological roles of Gcr1^U^ and Gcr1^S^ isoforms

Although we elucidated that Gcr1^U^ and Gcr1^S^ form distinct types of complexes, it is not yet clear why cells have both forms of Gcr1 complex. In line with the normal growth phenotypes of the Gcr1^U^ and Gcr1^S^ strains, ChIP-seq experiments revealed that the binding targets of Gcr1^U^ and Gcr1 ^S^ are almost identical. However, each Gcr1 complex may differ in DNA binding activity, transcriptional activation activity, or interaction with other transcription factors. Because the level of spliced *GCR1* mRNA producing Gcr1^S^ increased at later growth phase, Gcr1^S^ and Gcr1^U^ might have diverse roles depending on the growth phase (16). In fact, we observed some growth-dependent divergences between Gcr1^S^ and Gcr1^U^ in terms of target gene expression and DNA binding. Although expression of glycolytic genes decreased after diauxic shift, Gcr1^S^ DNA binding increased upon diauxic shift, exhibiting the opposite trend as compared with Gcr1^U^. In addition, the Gcr1^S^ strain showed higher expression levels of glycolytic genes than the Gcr1^U^ strain, especially after a diauxic shift. Considering the similar growth rates of the Gcr1^S^ and Gcr1^U^ strains, the observed differences between Gcr1^S^ and Gcr1^U^ might not be critical for cell growth at least under our culture conditions. These results are consistent with the fact that most of introns in *S. cerevisiae* can be deleted without any growth defects under normal conditions, but several intron deletions cause minor phenotypes under specific growth conditions (33). Therefore, in wild type cells producing both isoforms, changes in the ratio of Gcr1^U^ and Gcr1^S^ isoforms might contribute to sophisticated regulation of cell growth depending on environmental conditions. It needs further study to understand how the splicing is regulated and what the specific roles of the two types of Gcr1 complex are.

The Gcr1^S^ΔLZ1 strain which produced an inactive Gcr1^S^ monomer revealed a unique phenotype of respiration defect involving the failure of respiratory genes induction after diauxic shift. Although most of the respiratory genes are not direct targets of Gcr1, we detected direct binding of the Gcr1^S^ monomer (Gcr1^S^ co-expressed with the USS domain) to the *ALD4* promoter at the diauxic shift phase. Furthermore, the respiratory defect of Gcr1^S^ΔLZ1 strain was restored by overexpressing *ALD4* from a heterologous promoter, suggesting that inactivation of *ALD4* induction may be a major reason for the respiratory defect in the Gcr1^S^ΔLZ1 strain. It is unclear how the binding of the Gcr1^S^ monomer to the *ALD4* promoter inhibits transcription, but it could be through inhibition of other essential transcription factors. Considering that *GCR1* deletion does not affect respiratory growth, Gcr1 is unnecessary for activation of the respiratory genes after diauxic shift. However, the dominant negative effect of the Gcr1^S^ monomer on respiratory growth suggests a potential regulatory role for Gcr1 isoforms in transition from fermentative to respiratory growth. Based on the fact that overexpression of a single *ALD4* gene was enough to restore the respiratory defect of the Gcr1^S^ΔLZ1 strain, global metabolic regulation could be achieved by fine-tuning a few essential target genes.

### Regulation of *GCR1* by splicing

The *GCR1* gene has an unusually long intron of 739 nucleotides and produces multiple spliced isoforms by alternative splicing (15,16,18). The intron-containing genes in *S. cerevisiae* comprise only about 5% of the genome (34). Although alternative splicing is extensively used in metazoans to increase proteome diversity from a single gene, there are few known examples of alternative splicing in *S. cerevisiae* producing functional proteins with different roles. Alternative splicing of *PTC7* (35) and *SRC1* (36) genes generate proteins with alternate cellular localizations (nuclear envelope or mitochondria) and assorted folding patterns in the membrane, respectively. In addition, mitochondrial genes have extremely complex splicing patterns among multiple introns, encoding various essential proteins related to the respiratory chain complex (37). *GCR1* is the first example of a gene producing two functionally diverse transcription factors by splicing and intron retention.

Many intron-containing genes in *S. cerevisiae* generate non-productive mRNA species containing premature termination codons (PTC) by alternative splicing, which are degraded by the nonsense-mediated mRNA decay (NMD) system (38). Therefore, alternative splicing in *S. cerevisiae* may serve to down-regulate gene expression during stress. In fact, the five alternatively spliced *GCR1* transcripts with the PTC were degraded by NMD, and heat shock affected the selection of splicing sites (15). In addition, un-spliced *GCR1* mRNA is also a target of NMD as a quality control system as observed for other intron-containing genes (15). Therefore, splicing may regulate *GCR1* at multiple levels including the formation of two functional proteins and the condition-specific degradation of mRNAs to suppress gene expression. Such complicated regulatory mechanisms of the Gcr1 transcription factor might reflect the importance of sophisticated regulation of glycolysis for the survival of *S. cerevisiae* in an ever-changing natural environment.

## DATA AVAILABILITY

The ChIP-seq and RNA-seq data have been deposited in the NCBI Sequence Read Archive (SRA) under the accession numbers of BioProject ID PRJNA639179 (SRA accession numbers: SRR12006023-SRR12006028 and SRR12006035-SRR12006036 for ChIP seq, SRR12006021-SRR12006022 and SRR12006029-SRR12006036 for RNA-seq).

## Supplementary Material

gkaa1221_Supplemental_Files
