# Supplementary material for: Differential activation mechanisms of two isoforms of Gcr1 transcription factor generated from spliced and un-spliced transcripts in Saccharomyces cerevisiae
Source: Nucleic Acids Res. 2020 Dec 24;49(2):745–59. doi: 10.1093/nar/gkaa1221 (PMC7826247; doi:10.1093/nar/gkaa1221)
Supplement: gkaa1221_Supplemental_Files [file gkaa1221_supplemental_files.zip › Cha et al _ Supplementary data NAR R1.pdf]

Supplementary data

Differential activation mechanisms of two isoforms of Gcr1 transcription factor generated from spliced and un-spliced transcripts in *Saccharomyces cerevisiae*

Seungwoo Cha[1], Chang Pyo Hong[2], Hyun Ah Kang[3], and Ji-Sook Hahn[1*]

[1]School of Chemical and Biological Engineering, Institute of Chemical Processes, Seoul National University, 1 Gwanak-ro, Gwanak-gu, Seoul 08826, Republic of Korea

[2]Theragen Bio Co., Ltd, 145 Gwanggyo-ro, Yeongtong-gu, Suwon-si, Gyeonggi-do 16229, Republic of Korea

[3]Department of Life Science College of Natural Science, Chung-Ang University, 84 Heukseok-ro, Dongjak-gu, Seoul 06974, Republic of Korea

[*]Corresponding author:

Phone: +82-2-880-9228

Fax: +82-2-888-1604

e-mail: hahnjs@snu.ac.kr

Supplementary Figures

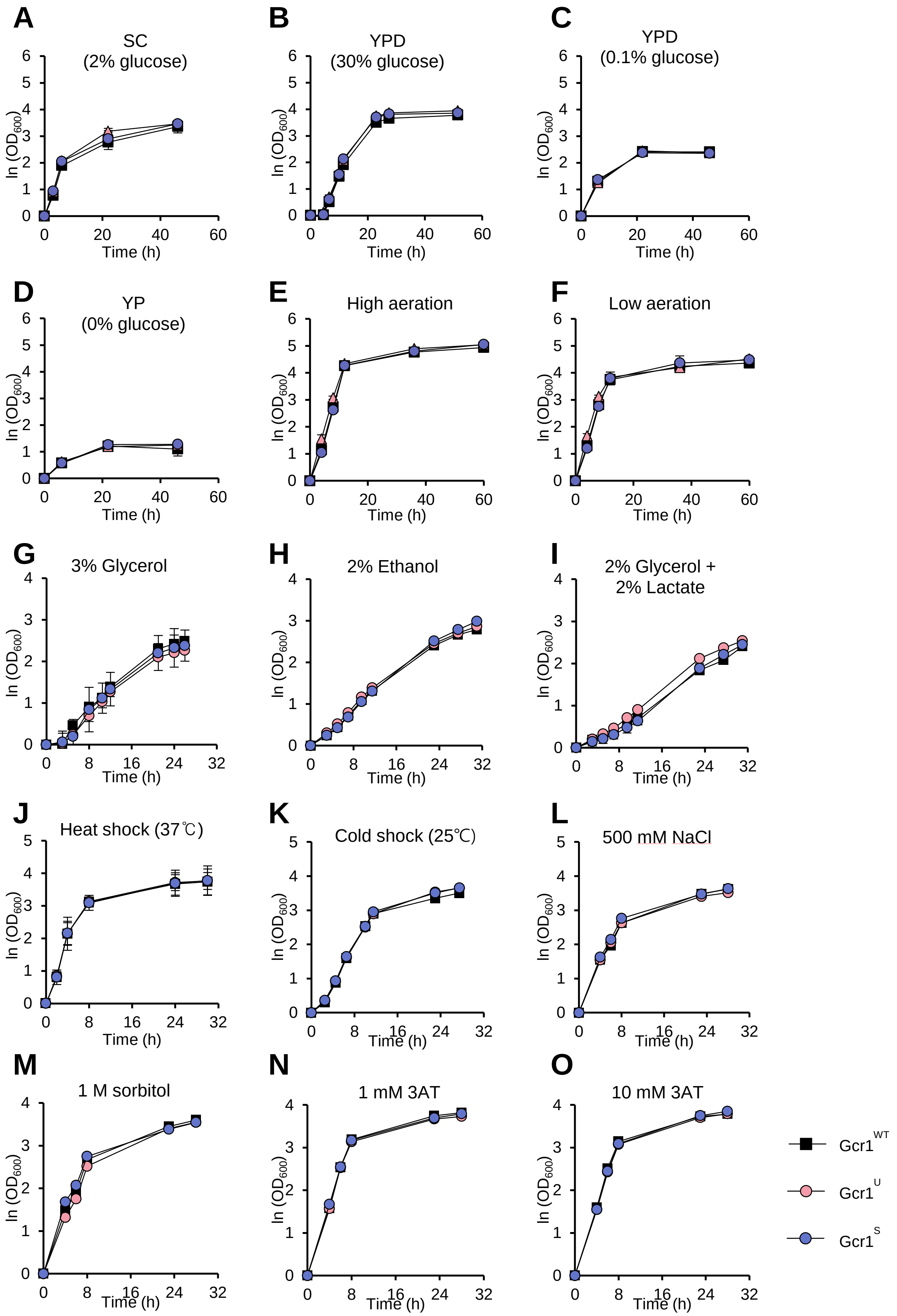

Figure S1. Growth curves of Gcr1^{WT}, Gcr1^{U}, and Gcr1^{S} strains under various stress conditions, related to Figure 1

Three strains were grown in SC media containing 2% glucose (A), YPD media containing high (B) or low (C) concentration of glucose, or no glucose (D), and high (E, 5 mL culture in 50 mL flask) or low (F, 50 mL culture in 50 mL flask) aeration. Different carbon sources (G, 3% glycerol; H, 2% ethanol; I, 2% glycerol + 2% lactate), temperature change (J, heat shock 37℃; K, cold shock 25℃), osmotic stresses (L, 500 mM NaCl; M, 1M sorbitol), and chemical stresses (N, 3-amino triazole 1 mM; O, 10 mM) were also tested. The error bars indicate the standard deviations of two independent experiments.

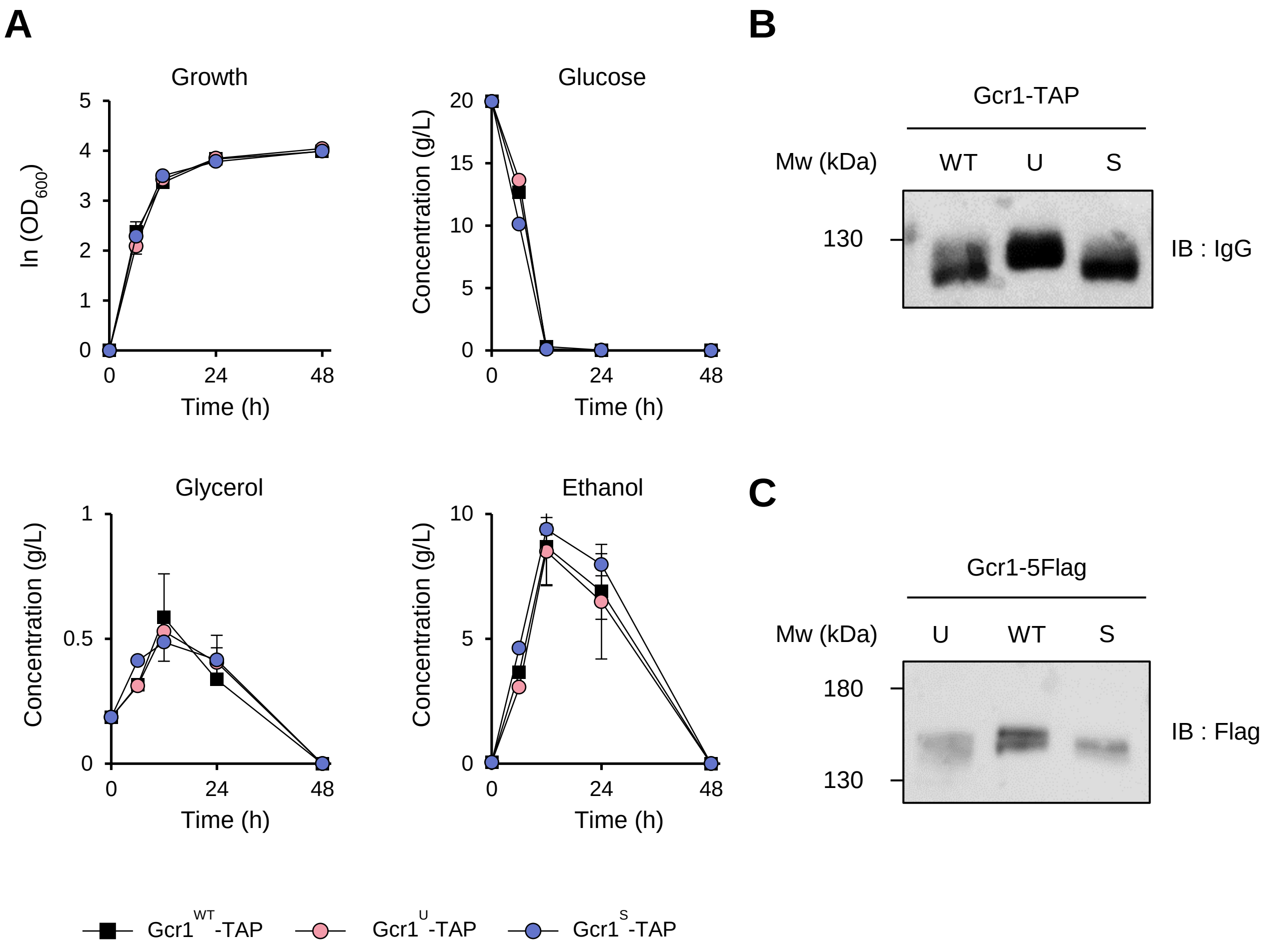

Figure S2. Properties of TAP-tagged Gcr1^{WT}, Gcr1^{U} and Gcr1^{S} strains, related to Figure 1

(A) Growth curves and metabolite profiles of strains expressing TAP-tagged Gcr1^{WT}, Gcr1^{U} and Gcr1^{S}. Error bars indicate standard deviations of three independent experiments.

(B, C) Immunoblotting analysis of TAP (B) or Flag (C) - tagged Gcr1 proteins. Strains expressing TAP or Flag-tagged Gcr1^{WT}, Gcr1^{U} and Gcr1^{S} were grown in YPD media until the exponential phase, and Gcr1-TAP or Gcr1-Flag proteins were detected by immunoblotting using anti-IgG or anti-Flag (DDDDK) antibody.

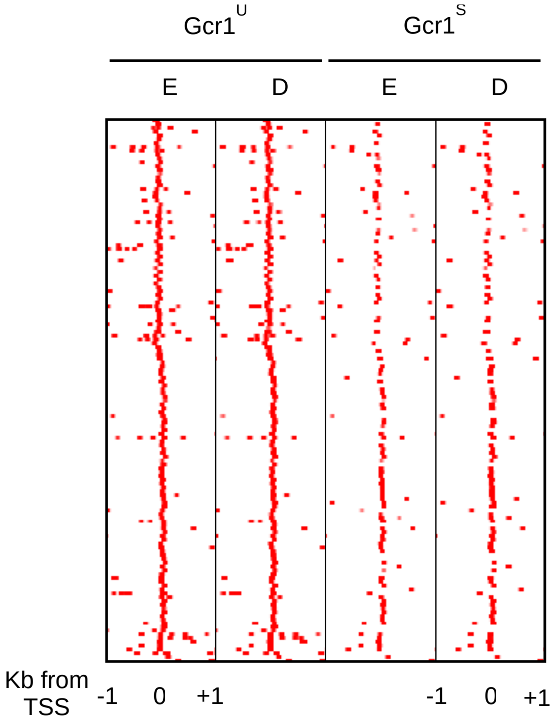

Figure S3. Heatmap of Gcr1^{U} and Gcr1^{S} occupancies from two independent ChIP-seqs at exponential phase and diauxic shift phases, related to Figure 2

Regions are sorted by the pattern of distribution of ChIP-seq reads mapped to the peaks of Gcr1^{U} and Gcr1^{S}. The x axis indicates Gcr1 occupied regions within 2 kb of gene TSS and the y axis indicates each gene. E, exponential phase; D, diauxic shift Phase.

Table S1

Strains used in this study

Strain	Description	Genotype	Reference
BY4741	Wild type	*S. cerevisiae MATa his3Δ1 leu2Δ0 met15Δ0 ura3Δ0*	EUROSCARF
JHY9000	*gcr1*Δ	BY4741 *gcr1*Δ	This study
JHY9001	*GCR1*ΔAH	BY4741 *GCR1* (Δ1223-1297)	This study
JHY9002	*GCR1*ΔLZ1	BY4741 *GCR1* (Δ1550-1639)	This study
JHY9003	*GCR1*ΔSP	BY4741 *GCR1* (Δ1676-2527)	This study
JHY9004	*GCR* ΔDBD	BY4741 *GCR1* (Δ2642-3106)	This study
JHY9005	*GCR1 gcr2*Δ	BY4741 *gcr2*Δ	This study
JHY9006	*GCR1 GCR2*Δ2H	BY4741 *GCR2* (Δ748-1440)	This study
JHY9007	*GCR1 GCR2*ΔLZ2	BY4741 *GCR2* (Δ1489-1602)	This study
JHY9100	*GCR1*^{U}	BY4741 *GCR1* (Δ1-574)	This study
JHY9101	*GCR1*^{U} ΔAH	JHY9100 *GCR1*^{U} (Δ649-723)	This study
JHY9102	*GCR1*^{U} ΔLZ1	JHY9100 *GCR1*^{U} (Δ976-1065)	This study
JHY9103	*GCR1*^{U} ΔSP	JHY9100 *GCR1*^{U} (Δ1102-1953)	This study
JHY9104	*GCR1*^{U} ΔDBD	JHY9100 *GCR1*^{U} (Δ2068-2532)	This study
JHY9105	*GCR1*^{U} *gcr2*Δ	JHY9100 *gcr2*Δ	This study
JHY9106	*GCR1*^{U} *GCR2*Δ2H	JHY9100 *GCR2* (Δ748-1440)	This study
JHY9107	*GCR1*^{U} *GCR2*ΔLZ2	JHY9100 *GCR2* (Δ1489-1602)	This study
JHY9200	*GCR1*^{S}	BY4741 *GCR1* (Δ4-742)	This study
JHY9201	*GCR1*^{S} ΔAH	JHY9200 *GCR1*^{S} (Δ484-558)	This study
JHY9202	*GCR1*^{S} ΔLZ1	JHY9200 *GCR1*^{S} (Δ811-900)	This study
JHY9202A	*GCR1*^{S} ΔLZ1+ ALD4	JHY9200 *GCR1*^{S} (Δ811-900) *ura3Δ0::*P$_{TEF1}$-*ALD4*-T$_{CYC1}$	This study
JHY9203	*GCR1*^{S} ΔSP	JHY9200 *GCR1*^{S} (Δ937-1788)	This study
JHY9204	*GCR1*^{S} ΔDBD	JHY9200 *GCR1*^{S} (Δ1903-2367)	This study
JHY9205	*GCR1*^{S} *gcr2*Δ	JHY9200 *gcr2*Δ	This study
JHY9206	*GCR1*^{S} *GCR2*Δ2H	JHY9200 *GCR2* (Δ748-1440)	This study
JHY9207	*GCR1*^{U} *GCR2*ΔLZ2	JHY9200 *GCR2* (Δ1489-1602)	This study
JHY9210	*GCR1*^{S}+USS	JHY9200 *ura3Δ0::*P$_{GCR1}$-*GCR1*^{U} (1-168)-T$_{GCR1}$	This study
JHY9211	*GCR1*^{S}+USS^{F12L}	JHY9200 *ura3Δ0::*P$_{GCR1}$-*GCR1*^{U} (1-168)^{T34C}-T$_{GCR1}$	This study
JHY9212	*GCR1*^{S}+USS^{L50P}	JHY9200 *ura3Δ0::*P$_{GCR1}$-*GCR1*^{U} (1-168)^{T149C}-T$_{GCR1}$	This study
JHY9210F	*GCR1*^{S}+USS-5Flag	JHY9200 *ura3Δ0::GCR1*^{U} (1-168)-*5Flag ::hphMX6*	This study
JHY9211F	*GCR1*^{S}+USS^{F12L}-5Flag	JHY9200 *ura3Δ0::GCR1*^{U} (1-168)^{T34C}-*5Flag ::hphMX6*	This study
JHY9212F	*GCR1*^{S}+USS^{L50P}-5Flag	JHY9200 *ura3Δ0::GCR1*^{U} (1-168)^{T149C}-*5Flag ::hphMX6*	This study

	GCR1-TAP	BY4741 *GCR1-TAP::his3MX6*	(Ghaemmaghami et al., 2003)
JHY9302	*GCR1-5Flag*	BY4741 *GCR1^{WT}-5Flag::hphMX6*	This study
JHY9310	*GCR1^{U}-TAP*	BY4741 *GCR1^{U}-TAP::his3MX6*	This study
JHY9311	*GCR1^{U}-TAP GCR2-5Flag*	JHY9310 *GCR2-5Flag::hphMX6*	This study
JHY9312	*GCR1^{U}-5Flag*	BY4741 *GCR1^{U}-5Flag::hphMX6*	This study
JHY9320	*GCR1^{S}-TAP*	BY4741 *GCR1^{S}-TAP::his3MX6*	This study
JHY9321	*GCR1^{S}-TAP GCR2-5Flag*	JHY9320 *GCR2-5Flag::hphMX6*	This study
JHY9322	*GCR1^{S}-5Flag*	BY4741 *GCR1^{S}-5Flag::hphMX6*	This study
JHY9322U1	*GCR1^{S}-5Flag* + USS	JHY9322 *ura3∆0::*P_{GCR1}-*GCR1^{U}* (1-168)-T_{GCR1}	This study

Table S2

Plasmids used in this study

Plasmid	Relevant characteristics	Reference
Plasmids for *S. cerevisiae*		
pFA6a-5Flag-hphMX6	Vector for genomic 5xFlag epitope tagging, hygromycin antibiotic marker	(Noguchi et al., 2008)
pRS413	CEN/ARS plasmid, *HIS3* marker	(Sikorski and Hieter, 1989)
p413Gcr1^{WT}	P_{GCR1}-*GCR1*-T_{GCR1} cloned between *Spe*I and *Xho*I sites of pRS413	This study
p413Gcr1^{U}	pRS413 containing P_{GCR1}-*GCR1* (Δ1-574)-T_{GCR1} generated by site directed mutagenesis of p413Gcr1^{WT}	This study
p413Gcr1^{S}	pRS413 containing P_{GCR1}-*GCR1* (Δ4-742)-T_{GCR1} generated by site directed mutagenesis of p413Gcr1^{WT}	This study
pRS416	CEN/ARS plasmid, *URA3* marker	(Sikorski and Hieter, 1989)
p416TEF	P_{TEF1} and T_{CYC1} cloned at *Sac*I/*Xba*I and *Xho*I/*Kpn*I sites, respectively, of pRS416	(Mumberg et al., 1995)
p416TEF-ALD4	*ALD4* cloned at *Spe*I/*Sal*I sites of p416TEF	This study
p416GCR1PT	P_{GCR1} and T_{GCR1} cloned at *Sac*I/*Xba*I and *Xho*I/*Kpn*I sites, respectively, of pRS416	This study
p416GCR1-USS	pRS416 containing P_{GCR1}-*GCR1*^{U}(1-168)-T_{GCR1} generated by cloning *GCR1*^{U} (1-168) between *Xba*I and *Xho*I sites of p416GCR1PT	This study
p416GCR1-USS^{F12L}	pRS416 containing P_{GCR1}-*GCR1*^{U}(1-168)^{T34C}-T_{GCR1} generated by directed mutagenesis of p416GCR1-USS	This study
p416GCR1-USS^{L50P}	pRS416 containing P_{GCR1}-*GCR1*^{U}(1-168)^{T149C}-T_{GCR1} generated by directed mutagenesis of p416GCR1-USS	This study
Plasmids for CRISPR/Cas9-mediated genome editing		
Coex413-Cas9	Coex413 containing P_{TDH3}-*Cas9*-T_{TPI1}	(Hong et al., 2019)
Coex413-Cas9-gGCR1	Coex413-Cas9 with gGCR1 gRNA	This study
Coex413-Cas9-gGCR1$^{U/S}$	Coex413-Cas9 with gGCR1$^{U/S}$ gRNA	This study
Coex413-Cas9-gGCR1AH	Coex413-Cas9 with gGCR1AH gRNA	This study
Coex413-Cas9-gGCR1SP	Coex413-Cas9 with gGCR1SP gRNA	This study
Coex413-Cas9-gGCR1DBD	Coex413-Cas9 with gGCR1DBD gRNA	This study
Coex413-Cas9-gGCR1USS	Coex413-Cas9 with gGCR1USS gRNA	This study
Coex413-Cas9-gGCR2	Coex413-Cas9 with gGCR2 gRNA	This study
Coex413-Cas9-gGCR2LZ	Coex413-Cas9 with gGCR2LZ gRNA	This study
Coex416-Cas9	Coex416 containing P_{TEF1}-*Cas9*-T_{TPI1}	This study
Coex416-Cas9-gURA3Δ0	Coex416-Cas9 with gURA3Δ0 gRNA	This study

Table S3

20-bp gRNA sequences used in this study

Name	gRNA sequence [a] (5’ to 3’)	Description
gGCR1	AATTAACTACAGAAAATATCagg	Used for deletion of *GCR1* and the leucine zipper domain of *GCR1*
gGCR1$^{U/S}$	TGCGTCTGTCTGCGTACAAGagg	Used for producing JHY9100, JHY9200 strains
gGCR1AH	TGCTTCAAATAAGACAGCCAtgg	Used for deletion of the alpha helix domain *GCR1*
gGCR1SP	TTGGGCTTGTCCGTTGGGCTtgg	Used for deletion of the serine-proline rich domain of *GCR1*
gGCR1DBD	TCCTACTAGAAGAATTATTAtgg	Used for deletion of the DNA binding domain of *GCR1*
gURA3Δ0	CGAGATTCCCGGGAGCTTTAtgg	Used for insertion at *ura3Δ0* site
gGCR1USS	CTTTTCAAGGGACAAATAACagg	Used for random mutagenesis of *GCR1*^{U} (1-168) in JHY9105
gGCR2	TGGCTAAAAATGCTAAAAATggg	Used for deletion of *GCR2* and the 2H domain of *GCR2*
gGCR2LZ	TCCTTAACCACTGCGTCTCTtgg	Used for deletion of the leucine zipper domain of *GCR2*

a. PAM sequences (NGG) are denoted as lower case letters.

Table S4

Primers used in this study [a, b]

Forward primer (5' to 3')	Reverse primer (5' to 3')	Usage
**Primer sequence for plasmid (for *S. cerevisiae*) generating**		
gcgACTAGTCCCGGATGAGAAACCTTTAAAATAAGCG	gcgCTCGAGCTGCTAGGTTTTTATATTGATTTTTGACAACAGAG	Cloning of Gcr1^{WT} using *Spe*I/*Xho*I
GCCTTTGATATATATTGAAAATGAATTTTCTGACTCAGGC	GCCTGAGTCAGAAAATTCATTTTCAATATATATCAAAGGC	Generation of p413Gcr1^{U} by site directed mutagenesis
TTTGATATATATTGAAAATGCAAACAAGTGTTGATAGTAC	GTACTATCAACACTTGTTTGCATTTTCAATATATATCAAA	Generation of p413Gcr1^{S} by site directed mutagenesis
gcgGAGCTCCCCGGATGAGAAACCTTTAAAATAAGCG	gcgTCTAGATTTCAATATATATCAAAGGCCAATTAAATATAACCGTCGTTTG	Cloning of P_{GCR1} using *Sac*I/*Xba*I
gcgCTCGAGGTTTATTGAGGTTGTCCGCGACAATAG	gcgGGTACCCTGCTAGGTTTTTATATTGATTTTTGACAACAGAG	Cloning of T_{GCR1} using *Xho*I/*Kpn*I
gcgTCTAGAATGAATTTTCTGACTCAGGCTATGTCAGAAAC	gcgCTCGAGTTACTGTTCCAATTGAGAAAGTAGGGCATTTAATTGG	Cloning of *GCR1*^{U} (1-168) using *Xba*I/*Xho*I
GAATTTTCTGACTCAGGCTATGTCAGAAACTCTTCAAGGGACAAATAACAGGATAAAACG	CGTTTTATCCTGTTATTTGTCCCTTGAAGAGTTTCTGACATAGCCTGAGTCAGAAAATTC	Generation of p416GCR1-USS^{F12L} by site directed mutagenesis
CTAACCAATTAAATGCCCCACTTTCTCAATTGG	CCAATTGAGAAAGTGGGGCATTTAATTGGTTAG	Generation of p416GCR1-USS^{L50P} by site directed mutagenesis
gcgACTAGTATGTTCAGTAGATCTACGCTCTGC	gcgGTCGACTTACTCGTCCAATTTGGCACGGAC	Cloning of *ALD4* into p416TEF using *Spe*I/*Sal*I
**Primer sequence for generating donor DNA by overlapping PCR**		
CAAGTGACAAACGACGGTTATATTTAATTGGCCTTTGATATATATTGAAAGTTTATTGAG	GCTTCGTTATTTTGTTGAAGGAACTATTGTCGCGGACAACCTCAATAAACTTTCAATATA	*gcr1* deletion
TTGAACCTTCTAAAGAGTTGATCGATTTGGTATTTCCATGGCTGTCTTATGGCAGGGTTG	AATAAGGGATGTTGACTCAGATATCGGAGATCCTGAATGGCAACCCTGCCATAAGACAGC	Deletion of the alpha helix domain of *GCR1*
AGGAAGTGAGTCAAAAAGTTGATTCTTACTTTATGGAATTATCAAAAAAACTGTTGCAGA	GACTTTGATCCGATCGCCTGATTTCCTGAAAGCAATTGTCTCTGCAACAGTTTTTTTGAT	Deletion of the leucine zipper domain of *GCR1*
GTAACCAAATTTTGCTGTTGCAGAGACAATTGCTTTCAGGAAATCAGGCGATTCATAATT	GTCTTTGTCACTGTACCACTTGAATTTGCAGCCTCAGTAGAATTATGAATCGCCTGATTT	Deletion of the serine-proline rich domain of *GCR1*
CGCAGAGTTCTTCTAAGTTTGAAATTATAAATAAAAAGGATACGAAGGCGTAAGTTTATT	TCGTTATTTTGTTGAAGGAACTATTGTCGCGGACAACCTCAATAAACTTACGCCTTCGTA	Deletion of the DNA binding domain of *GCR1*
AAGGAACCTGAGAACACAAAGAGTATTTGACGAAAAGTTACACTCACATACACGATAATA	CACCAGAAAATTAAAAGAGAAAGCAATATATGTTAAACATTATTATCGTGTATGTGAGTG	*gcr2* deletion

GACGCAGGAAGGGAAACTCCTTAAATACATCAACTAAAGGCTCCCCATCACCATTGAAAG	ATTCTTTGGCCTTTTTCAGAAATGATTGCGTCATAAGCTTCTTTCAATGGTGATGGGGAG	Deletion of the 2H domain of *GCR2*
GCCCATTGAAAGAAGCTTATGACGCAATCATTTCTGAAAAAGGCCAAAGATGACACGATA	CAGAAAATTAAAAGAGAAAGCAATATATGTTAAACATTATTATCGTGTCAAGGCCAAAGA	Deletion of the leucine zipper domain of *GCR2*
Primer sequence for generating donor DNA by PCR from cloned plasmids		
CAAGTGACAAACGACGGTTATATTTAATTG	TTATTTGTCCCTTGAAAAGTTTCTGACATAG	Generation of JHY9100 strain using p413Gcr1^{U} as a template
CAAGTGACAAACGACGGTTATATTTAATTG	ATGGAATAAAAGTTTGAGCTCGTGCTG	Generation of JHY9200 strain using p413Gcr1^{S} as a template
TTAATGTGGCTGTGGTTTCAGGGTCCATAAAGCTTCCCGGATGAGAAACCTTTAAAATAA	TTTAGTATACATGCATTTACTTATAATACAGTTTTCTGCTAGGTTTTTATATTGATTTTT	*ura3Δ0* site insertion with P_{GCR1}, T_{GCR1}
TTAATGTGGCTGTGGTTTCAGGGTCCATAAAGCTTATAGCTTCAAAATGTTTCTACTCC	TTTAGTATACATGCATTTACTTATAATACAGTTTTGCAAATTAAAGCCTTCGAGCGTCCC	*ura3Δ0* site insertion with P_{TEF1}, T_{CYC1}
Primer sequence for epitope tag for *S. cerevisiae*		
GAAGAGAAATTAAAGTATTGCAAAAGGCGACATAATACACCATCTCGGATCCCCGGGTTAATTAA	GTTTAAACGAGCTCGAATTCGTTTATTGAGGTTGTCCGCGACAATAGTTCCTTCAACAAAATAAC	Generation of C-terminal epitope tag at *GCR1*
GTTGTGTTAGAAGTATGTTAAGGGATTTACAAAGACGGATCCCCGGGTTAATTAA	AGAGAAAGCAATATATGTTAAACATTATTATCGTGGAATTCGAGCTCGTTTAAAC	Generation of C-terminal epitope tag at *GCR2*
CTAACCAATTAAATGCCCTACTTTCTCAATTGGAACAGCGGATCCCCGGGTTAATTAA	CTATTGTCGCGGACAACCTCAATAAACCTCGAGTTAGAATTCGAGCTCGTTTAAAC	Generating JHY9200U1, JHY9200U2 from JHY9200
CTAACCAATTAAATGCCCCACTTTCTCAATTGGAACAGCGGATCCCCGGGTTAATTAA	CTATTGTCGCGGACAACCTCAATAAACCTCGAGTTAGAATTCGAGCTCGTTTAAAC	Generating JHY9200U3 from JHY9200

[a] Restriction enzyme sites are underlined.

[b] Mutated nucleotides are shown in red.

Table S5

Primers for qRT-PCR and ChIP, related to Figure 3C, Figure 5, Figure 6A

Forward primer (5' to 3')	Reverse primer (5' to 3')	Target gene or locus
Primer sequence for qRT-PCR		
GCTGCTGGTAACACCGTCATCATTGG	CCACCACCAGTAGAGACATGGGAG	*PGK1*
CTGCTCAAGACTCTTTCGCTGCCAAC	CCGGCGTAGACAGCCTTGTCACCC	*ENO2*
CGAAAAGGAACCTGTCTCTGACTGGAC	CGGTAGAGACTTGCAAAGTGTTGGAGTGACC	*PYK1* (*CDC19*)
GCCAGCTGGTGCCAAGTGTTGT	TCCATCTTTTCGTAAATTTCTGGCAAGGTA	*ADH1*
GCCAGCCGGTGCAAAGTGCTCC	CTCCATCTTTTCGTAAATTTCTGGTAAACTG	*ADH2*
GAGCACAGGTTTGAAGGTGGCCAAG	GACTTCTTCACCCATTTCTCTACCGTAAC	*ALD6*
GGGTTGGCTGCTGGTATTCACACCT	CAGACATTTCCCTGCCCAAACCA	*ALD4*
GGATGTGAACGAGCGCCCATTATG	CCACGGCAACTTCCCAATACTTC	*GUT1*
Primer sequence for ChIP		
TCGGATCCTCAAAACCCTTAAAAAC	CGTGGCGGGTAAAGAAGAAAATGG	Promoter of *ACT1* (negative control)
GCGGGAAAGGGTTTAGTACCACATG	TGTCACACGATTCGGACAATTCTG	Promoter of *PGK1*
GGTACGGCTGTTATCCAGCGATGC	GTCAATTGTCACCGACAAACCCCCC	Promoter of *ENO2*
CGGATATCCTTTTGTTGTTTCCGG	GGGAGACCAACGAAGGTATTATAG	Promoter of *ADH*1
CAGGAATGTTCCACGTGAAGC	CAATGAGCTCTGAAGACGAATTG	Promoter of *ADH*2
CAAGACTTTTAGAACGGATAAGGTG	CCGGCCACAACTCAAACCAC	Promoter of *GUT1*
GGTACGGCTGTTATCCAGCGATGC	GTCAATTGTCACCGACAAACCCCCC	Promoter of *ALD4*

Table S6~S8 are provided in excel files.

Table S6. The list of gene IDs, gene names and clusters used for heatmap of Gcr1 ChIP-seq analysis, related to Figure 1C and Figure S3

Table S7. The list of ChIP-seq target genes of $Gcr1^{U}$ (U) and $Gcr1^{S}$ (S) at exponential (E) and diauxic shift (D) phases, related to Figure 1C

Table S8. The list of filtered target genes of $Gcr1^{U}$ (U) and $Gcr1^{S}$ (S) at exponential (E) and diauxic shift (D) phases, related to Figure 1C and D
